# Screening of Potential Biomarkers in the Peripheral Serum for Steroid-Induced Osteonecrosis of the Femoral Head Based on WGCNA and Machine Learning Algorithms

**DOI:** 10.1155/2022/2639470

**Published:** 2022-02-10

**Authors:** Jian Zhang, Chi Huang, Zehan Liu, Shuai Ren, Zilong Shen, Kecheng Han, Weiguang Xin, Guanyi He, Jianyu Liu

**Affiliations:** ^1^Department of Orthopedics, The Second Affiliated Hospital of Harbin Medical University, Harbin, 150001 Heilongjiang, China; ^2^Department of Orthopedics, The Fifth Hospital of Harbin, Harbin, 150040 Heilongjiang, China; ^3^Department of Orthopedics, The Second Hospital of Heilongjiang Province, Harbin, 150000 Heilongjiang, China

## Abstract

**Background:**

Steroid-induced osteonecrosis of the femoral head (SONFH) has produced a substantial burden of medical and social experience. However, the current diagnosis is still limited. Thus, this study is aimed at identifying potential biomarkers in the peripheral serum of patients with SONFH.

**Methods:**

The expression profile data of SONFH (number: GSE123568) was acquired from the Gene Expression Omnibus (GEO) database. Differentially expressed genes (DEGs) in SONFH were identified and used for weighted gene coexpression network analysis (WGCNA). Gene Ontology (GO) and Kyoto Encyclopedia of Genes and Genomes (KEGG) enrichment analyses were performed to investigate the biological functions. The protein-protein interaction (PPI) network and machine learning algorithms were employed to screen for potential biomarkers. Gene set enrichment analysis (GSEA), transcription factor (TF) enrichment analysis, and competing endogenous RNA (ceRNA) network were used to determine the biological functions and regulatory mechanisms of the potential biomarkers.

**Results:**

A total of 562 DEGs, including 318 upregulated and 244 downregulated genes, were identified between SONFH and control samples, and 94 target genes were screened based on WGCNA. Moreover, biological function analysis suggested that target genes were mainly involved in erythrocyte differentiation, homeostasis and development, and myeloid cell homeostasis and development. Furthermore, GYPA, TMCC2, and BPGM were identified as potential biomarkers in the peripheral serum of patients with SONFH based on machine learning algorithms and showed good diagnostic values. GSEA revealed that GYPA, TMCC2, and BPGM were mainly involved in immune-related biological processes (BPs) and signaling pathways. Finally, we found that GYPA might be regulated by hsa-miR-3137 and that BPGM might be regulated by hsa-miR-340-3p.

**Conclusion:**

GYPA, TMCC2, and BPGM are potential biomarkers in the peripheral serum of patients with SONFH and might affect SONFH by regulating erythrocytes and immunity.

## 1. Introduction

Steroid-induced osteonecrosis of the femoral head (SONFH), a chronic and progressive femoral head disease mainly induced by long-term exposure to excessive glucocorticoids, can cause hip joint damage and dysfunction and ultimately affect the quality of life [1]. The worldwide morbidity of SONFH is increasing year by year, and it is estimated that 20,000-30,000 patients are diagnosed with SONFH that does not depend on transmission each year in United States [2]. In particular, it is expected that the incidence of SONFH will rise in the next few years around the world because of the continuing impacts of the novel coronavirus pandemic since 2019 (COVID-19) [3]. Currently, although the diagnosis of SONFH, especially joint imaging techniques, is well established, patients with an early stage of SONFH are difficult to find because of the lack of effective and specific biomarkers [4]. Moreover, the cost and inconvenience of joint imaging techniques severely limit their application, specifically in China [5, 6]. Furthermore, total hip arthroplasty is effective in improving the quality of life, but most patients still endure the mental and financial pressure [7]. Therefore, the development of new biomarkers for SONFH diagnosis and treatment is urgently needed.

With the ongoing progression in sequencing technologies, bioinformatics analysis is an emerging and promising tool for screening potential biomarkers in a variety of neoplastic and nonneoplastic diseases [8, 9]. Data remining by using bioinformatics analysis based on public databases has facilitated the screening of new biomarkers for nonneoplastic diseases [10, 11]. In particular, combining weighted gene coexpression network analysis (WGCNA) and machine learning algorithms has greatly improved the accurate identification of disease-related biomarkers [12–15]. For example, FADD, CXCL2, and CXCL8 were identified as immune-related biomarkers of rheumatoid arthritis by integrating WGCNA and least absolute shrinkage and selection operator (LASSO) logistic regression and support vector machine recursive feature elimination (SVM-RFE) algorithms [12]. MACROD1 was found to contribute to the early diagnosis of tendinopathy based on the LASSO model, SVM-REF, and Gaussian mixture model (GMMs) algorithms [13]. Moreover, LSP1, GNLY, and MEOX2 are likely to aid in the diagnosis and treatment of rheumatoid arthritis based on WGCNA machine learning strategies. Furthermore, CDK1, TOP2A, ADRA1A, FANCI, XRCC1, TPX2, CCNB2, CDK4, GLYATL1, and CFHR3 were identified to be core hub genes as potential biomarkers and treatment targets for hepatoblastoma by integrating WGCNA and random forest (RF) algorithm [14]. However, most bioinformatics analyses for SONFH have not been performed by integrating WGCNA and machine learning algorithms.

Therefore, the present study is aimed at identifying potential biomarkers in the peripheral serum for SONFH by integrating WGCNA and machine learning algorithms, including LASSO logistic regression, SVM-RFE, and RF. First, we identified differentially expressed genes (DEGs) between SONFH and control samples in the GSE123568 dataset downloaded from the Gene Expression Omnibus (GEO) database (https://www.ncbi.nlm.nih.gov/geo/) in NCBI. Moreover, WGCNA was performed based on the expression matrix of DEGs to screen target genes, and machine learning algorithms were used to identify potential biomarkers. Finally, we investigated the possible functions and regulatory mechanisms of the potential biomarkers. This study may provide new biomarkers for the diagnosis and treatment of SONFH and contribute to clarifying the pathogenesis of SONFH.

## 2. Material and Methods

### 2.1. Data Collection and Preprocessing

The gene expression matrix of the peripheral serum for 30 SONFH and 10 control samples from China in the GSE123568 dataset, which was sequenced using the GPL15207 platform, was obtained from the GEO database.

### 2.2. Identification of DEGs in the Peripheral Serum of Patients with SONFH

“Limma” R package was used to screen DEGs between SONFH and control samples [16], and genes with *P* < 0.01 and ∣log_2_^FC^ | >1 were regarded as DEGs.

### 2.3. Screening of Target Modules and Genes Based on WGCNA

To screen potential genes associated with SONFH, the expression matrix of DEGs was used to create a weighted gene coexpression network by using “WGCNA” R package [17]. First, clustering of all samples was performed to guarantee a reliable network. Second, we calculated the Pearson correlation coefficient between each pair of genes to evaluate the expression similarity of genes and acquire a correlation matrix. Moreover, we used the soft threshold function to convert the correlation matrix into a weighted neighborhood matrix. To ensure that gene correlations were maximally consistent with scale-free distribution, we used a soft connectivity algorithm to select the optimal soft threshold. Subsequently, the neighborhood matrix was transformed into a topological overlap matrix (TOM). Furthermore, coexpression modules were obtained based on the criteria of dynamic tree cutting by setting the minimum number of genes in a module as 30. Finally, key modules were selected by correlation analysis, and genes in the key modules were considered as key genes.

### 2.4. Gene Ontology (GO) and Kyoto Encyclopedia of Genes and Genomes (KEGG) Enrichment Analyses

Biological function enrichment of GO and KEGG analyses was performed by using the “clusterProfiler” R package [18]. GO enrichment analysis was performed to investigate the gene-related biological process (BP), molecular functions (MF), and cellular components (CC). KEGG enrichment analysis was conducted to explore gene-related signaling pathways. Statistical significance was set at adjusted *P* value < 0.05.

### 2.5. Construction of a Protein-Protein Interaction (PPI) Network

A PPI network was constructed to investigate the protein interactions of genes through the Search Tool for the Retrieval of Interacting Genes (STRING, https://string-db.org/). Moreover, Cytoscape was selected to visualize the network, and the confidence score was set at 0.4.

### 2.6. Identification of Potential Biomarkers in the Peripheral Serum for SONFH Based on Machine Learning Algorithms

To begin with, the LASSO logistic regression algorithm [19] was performed to screen potential genes by using the “glmnet” R package [20], and receiver operating characteristic (ROC) analysis was selected to test the model reliability by calculating the area under the curve (AUC) value through the “pROC” R package [21]. Next, the SVM-RFE algorithm [22] was used to screen potential genes by using “e1071” R package [23]. In addition, the random forest (RF) algorithm [24] was conducted to screen potential genes by using the “randomForest” R package [25]. Similarly, the ROC curve was used to test the model reliability by using the “pROC” R package [21], and the top 10 genes based on %IncMSE ranking were regarded as the potential genes. Finally, overlapping genes among potential genes generated via LASSO, SVM-RFE, and RF algorithms were considered as potential biomarkers in the peripheral serum for SONFH.

### 2.7. Evaluation of the Expression Levels and Diagnostic Implications for Potential Biomarkers

Wilcoxon's rank-sum test was used to analyze the expression levels of potential biomarkers, and ROC analysis was performed to evaluate whether potential biomarkers could differentiate SNOFH samples from control samples by using the “pROC” R package [21].

### 2.8. Biological Functions and Regulating Mechanisms of Potential Biomarkers

Firstly, Gene Set Enrichment Analysis (GSEA) was performed using the “clusterProfiler” R package [18] to investigate the biological functions of potential biomarkers by the ordered gene expression matrix based on the Pearson correlation between each biomarker and other genes. Moreover, we also performed transcription factor (TF) enrichment analysis for potential biomarkers using the ChIP-X Enrichment Analysis 3 (ChEA3) database (https://amp.pharm.mssm.edu/chea3/). We also constructed a competing endogenous RNA (ceRNA) network by predicting miRNAs and lncRNAs as potential biomarkers in the miRwalk and miRanda databases, separately.

## 3. Results

### 3.1. Identification of DEGs in the Peripheral Serum of Patients with SONFH

**Figure 1:**
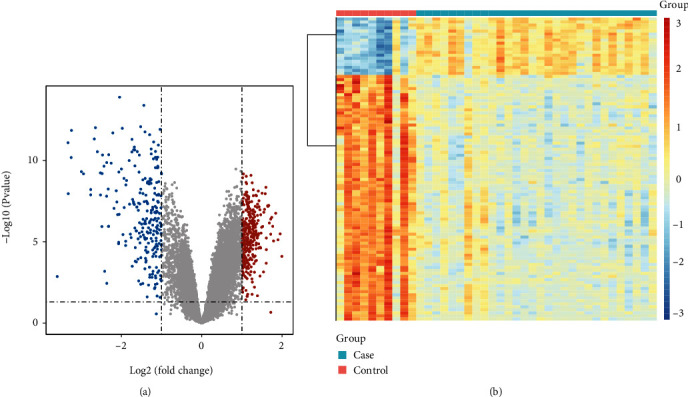
DEGs between SONFH patients and control samples. (a) Volcano plot showing the expression levels of DEGs. Red dots indicate upregulated genes in SONFH patients compared with control samples, while blue dots indicate downregulated genes in SONFH patients compared with control samples and gray dots indicate nonsignificantly different genes between SONFH patients and control samples. (b) Heat map showing the expression levels of top100 DEGs. Red indicates high expression, while blue indicates low expression.

By setting the cut-off value as *P* < 0.01 and ∣log_2_FC | >1, a total of 562 DEGs, including 318 upregulated and 244 downregulated genes, were identified in the peripheral serum of SONFH patients compared with control samples (Figures 1(a) and 1(b)).

### 3.2. Screening of Target Modules and Genes Based on WGCNA

**Figure 2:**
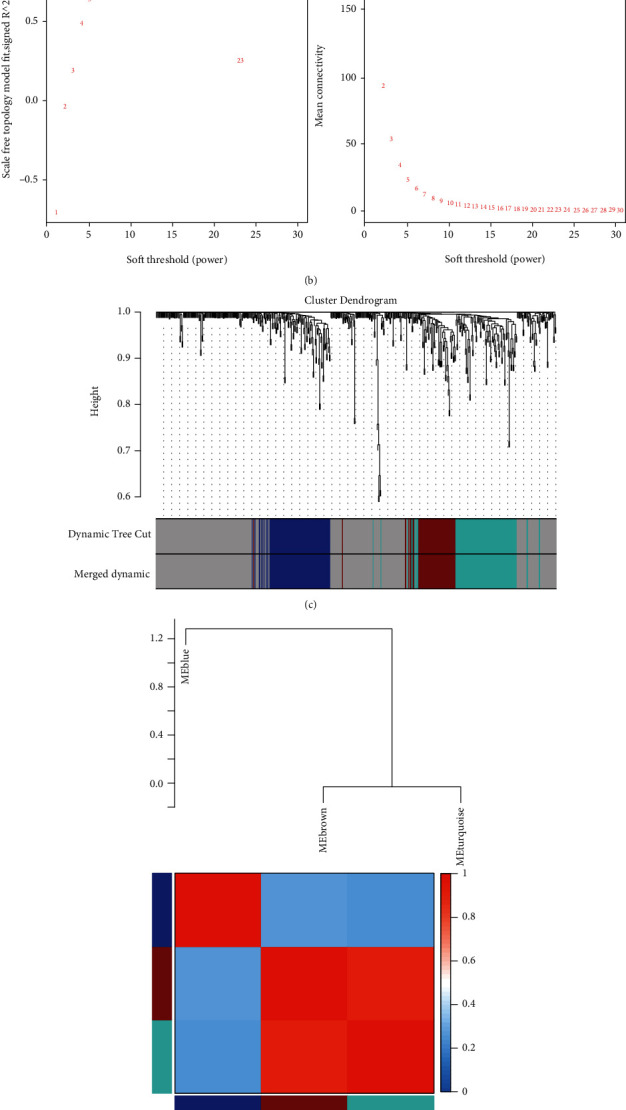
Identification of target genes by WGCNA. (a) Sample clustering analysis revealed that GSM3507251 sample was an outlier. (b) Soft threshold analysis suggested that gene associations were maximally consistent with the scale-free distribution and when *β* = 26. (c) Modules identified by merging modules with feature factors greater than 0.5 and setting the minimum number of genes in a module as 30. (d) Module correlations among the three identified modules. (e) Correlation between modules and SONFH.

To further screen genes related to SONFH, WGCNA was performed using 562 DEGs. As shown in Figure 2(a), clustering analysis of all samples showed that the GSM3507251 sample was poorly clustered. Therefore, this sample was excluded as an outlier in the WGCNA analysis. Next, the expression matrix of DEGs in the remaining 39 samples was used to construct a weighted gene coexpression network. The analysis of soft threshold selection revealed that gene associations were maximally consistent with the scale-free distribution and when *β* = 26 (scale free *R*^2^ = 0.85, Figure 2(b)). Moreover, three coexpression modules were screened in the weighted gene coexpression network by merging modules with feature factors greater than 0.5 and setting the minimum number of genes in a module to 30 (Figure 2(c)). Furthermore, we also investigated the module correlations and found that MEbrown and MEturquoise presented stronger correlations than MEblue (Figure 2(d)). Finally, the MEblue module was selected as the target module because it had the highest correlation with SONFH (Figure 2(e)), and a total of 94 DEGs in the MEblue module were regarded as target genes.

### 3.3. GO and KEGG Enrichment Analyses

**Figure 3:**
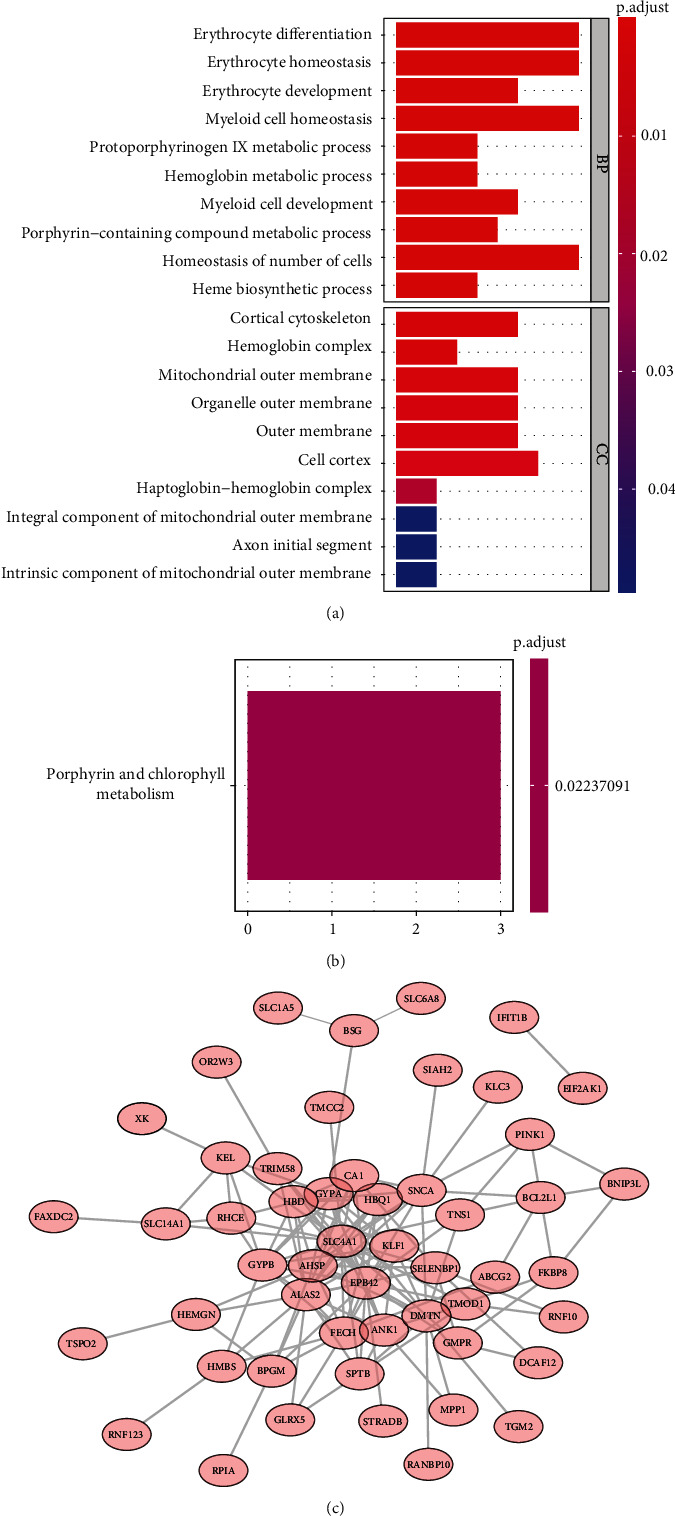
Functional enrichment analysis and PPI network: (a) GO results for the target genes; (b) KEGG results for the target genes; (c) PPI network of the target genes.

GO and KEGG enrichment analyses were performed to investigate the biological functions of the 94 target genes. GO analysis suggested that these target genes were mainly involved in erythrocyte differentiation, homeostasis, and development; myeloid cell homeostasis and development; and porphyrin metabolism-related BPs (Figure 3(a)). Moreover, these target genes were mainly associated with the cell cortex, cortical cytoskeleton, and mitochondrial and organelle outer membrane-related CCs (Figure 3(a)). Furthermore, KEGG enrichment analysis suggested that these target genes were enriched only in the porphyrin- and chlorophyll metabolism-related signaling pathways (Figure 3(b)).

### 3.4. Construction of a PPI Network

To further investigate the protein interactions of the target genes, we constructed a PPI network. As shown in Figure 3(c), the PPI network included 52 nodes with 146 edges, and EBP42, ALAS2, FECH, TMOD1, ANK1, SLC4A1, HBQ1, GYPA, KLF1, and DMTN could affect more proteins. Thus, these 52 target genes were retained for subsequent analyses.

### 3.5. Identification of Potential Biomarkers in the Peripheral Serum for SONFH Based on Machine Learning Algorithms

**Figure 4:**
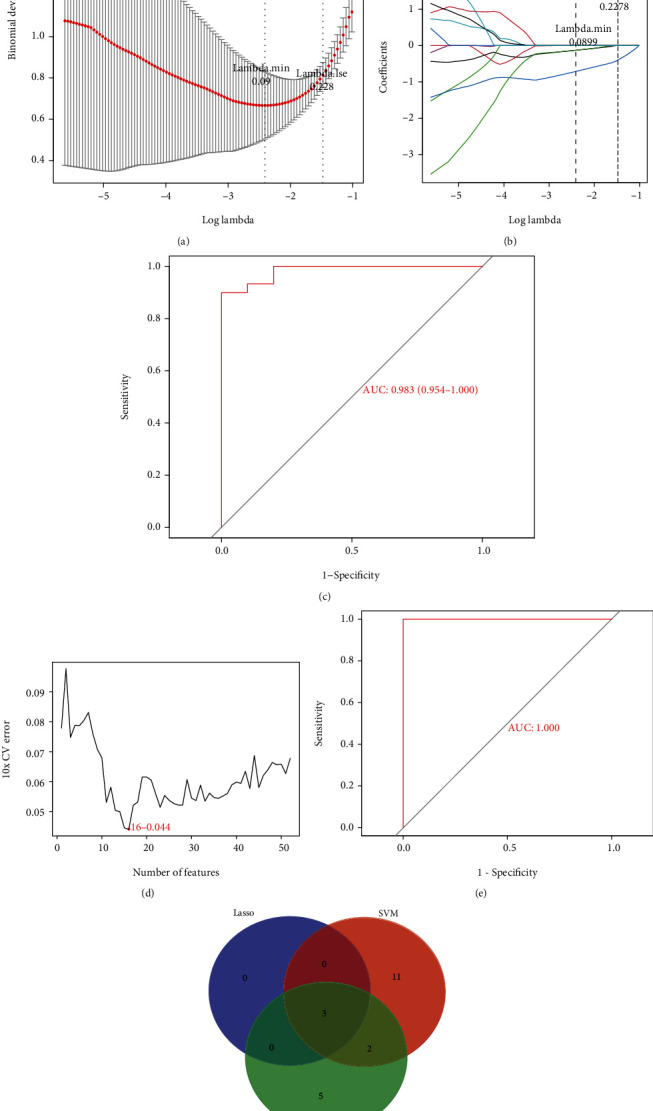
Identification of potential biomarkers for SONFH based on machine learning algorithms. (a) Log (Lambda) value of the three genes in LASSO model. (b) The most proper log (Lambda) value in LASSO model. (c) ROC curve of the LASSO model based on three genes. (d) The optimum error rate of SVM model based on 16 characteristic genes. (e) ROC curve of the RF module based on top 10 genes. (f) Venn diagram shows the overlapping genes in LASSO, SVM, and RF module.

To further identify the potential biomarkers in the peripheral serum of SONFH from 52 target genes, machine learning algorithms were selected and executed. Firstly, while constructing the LASSO model based on SONFH and control samples, *λ* analysis suggested that the model could accurately predict SONFH with *λ* = 3 (Figure 4(a)). Thus, GYPA, TMCC2, and BPGM were identified to build the LASSO module. We acquired the LASSO coefficient spectrum of the potential genes according to *λ* = 3 (Figure 4(b)). In addition, AUC analysis suggested that the LASSO module based on GYPA, TMCC2, and BPGM showed excellent performance (Figure 4(c)). On the other hand, SVM-RFE analysis revealed that the SVM model based on 16 characteristic genes showed an optimum error rate (0.044, Figure 4(d)). Thus, ANK1, BCL2L1, BPGM, BSG, FAXDC2, GYPA, HBQ1, HEMGN, IFIT1B, KEL, MPP1, SLC1A5, SPTB, TMCC2, TNS1, and TSPO2 were identified as the potential genes. At the same time, the RF algorithm identified the top 10 genes, including GYPA, RNF10, FECH, DMTN, FKBP8, BPGM, HEMGN, BNIP3L, and IFIT1B, from 52 target genes, and the RF module based on these 10 genes also showed good generalization performance (Figure 4(e)). Finally, three common potential genes, namely, GYPA, TMCC2, and BPGM, were regarded as the potential biomarkers in the peripheral serum of SONFH patients using the above three algorithms (Figure 4(f)).

### 3.6. Evaluation of the Expression Levels and Diagnostic Implications for the Potential Biomarkers

**Figure 5:**
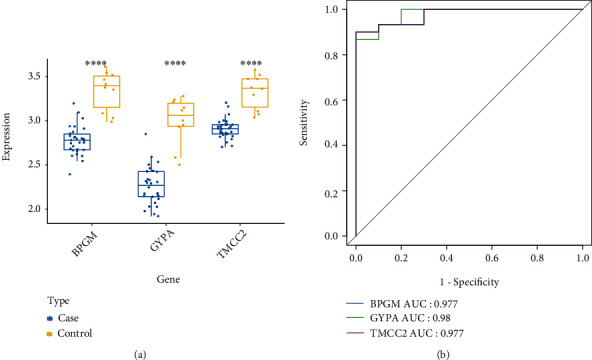
The expression levels and diagnostic implications of the potential biomarkers. (a) The expression levels of GYPA, TMCC2, and BPGM in SONFH patients and control samples. (b) ROC curves of GYPA, TMCC2, and BPGM.

To further investigate the role of GYPA, TMCC2, and BPGM in SONFH, we first observed their expression levels in SONFH patients. Interestingly, we found that the expression of GYPA, TMCC2, and BPGM was downregulated in SONFH patients compared with the control samples (Figure 5(a)). Moreover, ROC analyses suggested that GYPA, TMCC2, and BPGM might be used as diagnostic biomarkers in the peripheral serum of SONFH patients (Figure 5(b)).

### 3.7. Biological Functions and Regulating Mechanisms of the Potential Biomarkers

**Figure 6:**
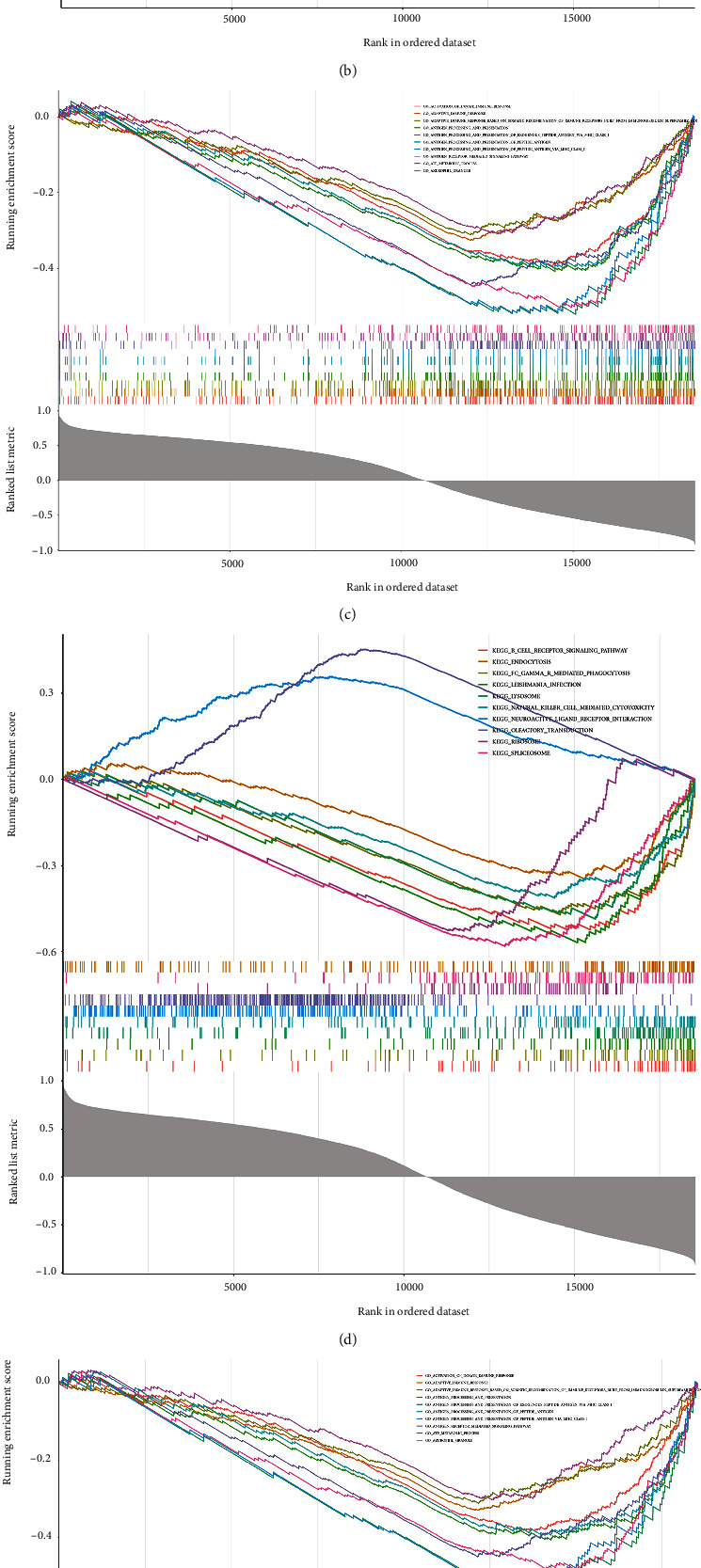
GSEA of potential biomarkers: (a) GO results for BPGM; (b) KEGG results for BPGM; (c) GO results for GYPA; (d) KEGG results for GYPA; (e) GO results for TMCC2; (f) KEGG results for TMCC2.

To further investigate the biological functions and regulatory mechanisms of GYPA, TMCC2, and BPGM, GSEA was performed based on their ordered gene expression matrix. As shown in Figure 6, GO and KEGG analyses revealed that GYPA, TMCC2, and BPGM are mainly involved in the B-cell receptor, endocytosis, FC gamma R-mediated D phagocytosis, T-cell, and natural killer cell-mediated cytotoxicity signaling pathways and are associated with activation of the innate immune response, adaptive immune response, antigen processing and presentation, antigen receptor signaling pathways mediated by antigen receptors, ATP metabolism, and B cell activation (Figure 6).

**Figure 7:**
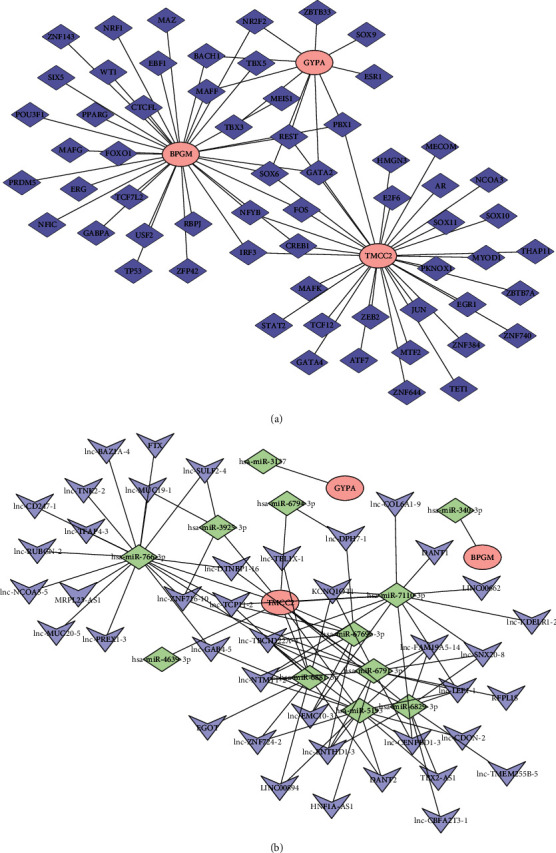
The regulating mechanisms of potential biomarkers. (a) The TF enrichment analysis of potential biomarkers. Blue diamonds indicate predicted TFs, and red ovals indicate potential biomarkers. (b) The ceRNA network of potential biomarkers. Red circles indicate potential biomarkers, green diamonds indicate miRNAs, and blue inverted cones indicate lncRNAs.

Moreover, to investigate the regulatory mechanisms of GYPA, TMCC2, and BPGM, we first predicted their TFs and constructed a TF-potential biomarker network (Figure 7(a)). Notably, GYPA might be regulated by ZBTB33, SOX9, ESR1, and BACH1; BPGM might be regulated by CTCFL, FOXO1, TCF7L2, USF2, RBPJ, and so on, and TMCC2 could be regulated by MAFK, JUN, SOX11, AR, and so on (Figure 7(a)). Furthermore, we also predicted the targeted miRNAs and lncRNAs of GYPA, TMCC2, and BPGM and constructed a ceRNA network (Figure 7(b)). Interestingly, only miRNAs were predicted for GYPA and BPGM, and the corresponding lncRNAs were not predicted (Figure 7(b)).

## 4. Discussion

SONFH is a complicated disease of the femoral head with complex pathogenesis, including genetic and environmental factors [26]. According to statistics, there are approximately 36,000 to 48,000 new SONFH patients in China each year [27, 28]. Currently, the clinical diagnosis of SONFH is sometimes full of significant difficulties due to the lack of frequent symptoms and valid diagnostic biomarkers, especially in the early stage of SONFH, and ultimately resulting in that most patients miss the best time for treatment and become difficult to reverse [29, 30]. Although total hip replacement is regarded as a proven treatment for femoral head necrosis, patients with SONFH are often too young to undergo total hip replacement. Thus, they still struggle with the psychological and economic burden of a revision surgery [31]. Therefore, identifying potential biomarkers is essential for the diagnosis of SONFH.

In the present study, we first identified 562 DEGs in the peripheral serum obtained from SONFH patients. Next, 94 genes of them were identified as target genes based on WGCNA. Interestingly, these 94 target genes were mostly related to erythrocyte differentiation, homeostasis, and development, myeloid cell homeostasis and development, and porphyrin metabolism-related BPs (Figure 3(a)). Thus, we speculated that these genes may play key roles in SONFH by regulating erythrocytes and myeloid cells. Recent studies have found that erythropoietin can protect rat models of SONFH by inhibiting the apoptosis of osteoblasts and osteocytes and increasing the expression of VEGF [32, 33]. In addition, increasing evidence has revealed that erythropoietin can promote bone repair in SONFH by regulating the hypoxia-inducible factor signaling pathway [34, 35]. Furthermore, erythropoietin also can prevent bone loss in mouse models of osteonecrosis of the femoral head by regulating osteogenesis, angiogenesis, and cell apoptosis [36]. On the other hand, we found that 94 target genes were enriched only in the porphyrin- and chlorophyll metabolism-related signaling pathways (Figure 3(b)). Clearly, metabolism is a key risk factor for nontraumatic SONFH [37, 38]. Therefore, our study may contribute to the understanding of the molecular mechanisms underlying SONFH.

Additionally, we further investigated the protein interactions among 94 target genes and screened 52 genes by constructing a PPI network. Finally, we identified GYPA, TMCC2, and BPGM as potential biomarkers using LASSO logistic regression, SVM-RFE, and RF algorithms. GYPA (Glycophorin A), a major sialoglycoprotein of the human erythrocyte membrane, has been found to be related to blood immunity by regulating the proliferation and differentiation of hematopoietic and immunocompetent cells in human marrow [39]. Thus, GYPA may play a critical role in SONFH by regulating blood immunity and red blood cells. TMCC2 (Transmembrane and Coiled-Coil Domain Family 2) has been revealed to be related to human erythroid differentiation [40]. Moreover, TMCC2 can affect A*β*PP metabolism [41] and nitrogen metabolism and excretion [42]. Hence, TMCC2 may play a key role in SONFH by affecting erythroid differentiation and metabolism. BPGM (Bisphosphoglycerate Mutase), a small molecule found at high concentrations in red blood cells where it binds to and decreases the oxygen affinity of hemoglobin, is associated with erythrocyte metabolic reprogramming in chronic kidney disease [43]. More importantly, BPGM is related to erythrocytosis [44, 45] and neutrophilia [46]. Therefore, BPGM may play a decisive role in SONFH by affecting erythroid proliferation and immunity. Notably, no studies have reported the role of GYPA, TMCC2, and BPGM in SONFH. Thus, further investigations are necessary.

Finally, we also investigated the biological functions and regulatory mechanisms of GYPA, TMCC2, and BPGM. Interestingly, GSEA revealed that GYPA, TMCC2, and BPGM were mainly involved in immune-related BPs and signaling pathways, such as T- and B-cell receptor signaling pathways. Currently, an increasing number of studies have shown that the immune response has a significant impact on the occurrence and development of SONFH. For example, it has been suggested that macrophages and CD4^+^ T cells are associated with SONFH [47, 48]. Therefore, GYPA, TMCC2, and BPGM may play key roles in SONFH by regulating the immunity. Moreover, we also explored the regulatory mechanisms of GYPA, TMCC2, and BPGM and found that GYPA might be regulated by hsa-miR-3137, and BPGM might be regulated by hsa-miR-340-3p. However, to the best of our knowledge, their regulatory mechanisms are rarely studied. Thus, further studies are required in the future.

## 5. Conclusion

In summary, 562 DEGs were screened between the peripheral serum of SONFH patients and control samples. Moreover, GYPA, TMCC2, and BPGM were identified as potential biomarkers in the peripheral serum of SONFH patients based on WGCNA and machine learning algorithms. Furthermore, we found that GYPA might be regulated by hsa-miR-3137 and that BPGM might be regulated by hsa-miR-340-3p. Therefore, our study may contribute to the understanding of SONFH and may help in improving the diagnosis of SONFH. However, further studies are needed to investigate the roles of GYPA, TMCC2, and BPGM.

## Data Availability

The GSE123568 dataset was obtained from the GEO database (https://www.ncbi.nlm.nih.gov/geo/).
